# A Survey of the Estimated Cost of Surgical Consumable Items Within Trauma and Orthopaedic Departments

**DOI:** 10.7759/cureus.63793

**Published:** 2024-07-04

**Authors:** Omkaar Divekar, Krushi Pandya, Anand B Divekar, Rahul Kanegaonkar

**Affiliations:** 1 Trauma and Orthopaedics, St. George's University, London, GBR; 2 Trauma and Orthopaedics, King's College London, London, GBR; 3 Trauma and Orthopaedics, William Harvey Hospital, Ashford, GBR; 4 Ear, Nose, Throat, Canterbury Christ Church University, Canterbury, GBR

**Keywords:** medical education, nhs england, orthopaedic surgery, cost reduction, healthcare expenditure

## Abstract

Introduction

The impact of the current economic and environmental climate, both nationally and globally, is further straining the NHS. This has led to scrutiny of high-expenditure areas, including consumables. Clinician’s knowledge surrounding health economics is sparse, and we conducted this survey to assess cost-awareness within the Trauma and Orthopaedic (T&O) departmental staff.

Methods

A questionnaire was digitally distributed to T&O staff in the East Kent Hospitals Trust. This included demographic data and to make estimations of the cost of 10 specialty-specific items. The data were analysed to determine the average, median, and interquartile range (IQR) of the estimated prices and compared to the actual costs.

Results

Approximately 7.1% of all item estimates were deemed ‘correct’. No correlation was seen between years of staff experience and the accuracy of estimates. ‘Kenalog 1 mL ampoule’ (Kenalog, Bristol-Myers Squibb, NJ) had the highest accuracy of estimation across all responses (13%), whilst both ‘kirschner wires’ and ‘3.2 drill bit’ had the lowest accuracy (4% each). The median estimated cost was closest to the actual cost for ‘cement pack’ (median estimate/actual cost = 0.9). The median estimated cost was furthest from the actual cost for ‘tourniquet cuffs’ (median estimate/actual cost = 0.16). ‘Velcro wrist splint’ was the item that was the most overestimated (median estimate/actual cost = 1.57), with only two of the 10 items being overestimated (‘velcro wrist splint’ and ‘dynamic hip screw and plate’). The most underestimated item was ‘tourniquet cuffs’ (median estimate/actual cost = 0.16).

Conclusions

There is a paucity of knowledge surrounding the cost of specialist T&O consumables. The limitations included the sample size (98 respondents) and geographical area (East Kent Hospitals Trust). This study shows that there is a need for further research into this topic, with long-term outcomes, which may be beneficial both economically and environmentally.

## Introduction

The impact of the current economic and environmental climate, both nationally and globally, is further straining an issue-saturated NHS [1]. With these rising concerns, interest has been generated surrounding high-expenditure specialities, namely surgical specialities and in particular Trauma and Orthopaedic (T&O) departments [2].

Operating theatres are acknowledged to be one of the largest expenditures within a hospital, with operating costs estimated to be approximately £1,200/hour [3]. Specifically, the use of sterilised surgical equipment has been long debated, with infection control being favoured over the fiscal and environmental impact of the implementation of disposable tools [4]. The risks to patients' health because of contamination have led to medical practice using single-use disposable equipment, increasing wastage. Studies have confirmed that the vast differences in equipment used from trust to trust, if more well-informed, may lead to a reduction in waste and expenditure [5].

This disparity has spurred a drive for medical schools across the country to embed sustainable healthcare teaching into their curricula, with a drive for high-quality care to prevent costly intervention in the future [6]. Whilst decision-making regarding waste reduction policies is centralised, all healthcare professionals have an ethical obligation to reduce their carbon footprint, whilst primarily providing an efficient and effective service [7].

This paper aims to further elucidate primarily the awareness of the cost of consumables within the T&O department and further ascertain if there are links between experience, geographical distribution, and job role. Furthermore, we have aimed to qualitatively identify whether T&O departments believe that the price of a consumable item should influence its use.

This article was previously presented as a poster at the Association of Surgeons in Training (ASIT) held in Bournemouth in March 2024.

## Materials and methods

This paper was approved by the Research and Innovation Department of East Kent Hospitals and contributed to a broader regional survey of NHS clinicians, varying in specialty. A digital questionnaire was generated using Microsoft Forms (Microsoft® Corp., Redmond, WA), including demographic data (e.g., age, gender, job title, years of experience). In the second section, participants were asked to estimate the cost of 10 general medical equipment items, followed by 10 T&O-specific consumables, in pounds and pence (Table 1). All items included in the survey had illustrations attached.

**Table 1:** List of trauma and orthopaedic-specific consumable items included in the survey

Item	Quantity	Actual Cost/£
Kenalog 1 mL ampoule	40 mg, box of 5	8.31
Kirschner wires, 1.6 mm	Box of 10	73.68
Standard size osteotome, 200 mm x 20 mm	One (1)	47.25
Bone nibbler	One (1)	90.56
Cement pack	40 grams	42.00
Tourniquet cuffs	Box of 10	144.18
3.2 mm drill bit	One (1)	28.00
Dynamic hip screw and plate	One (1)	44.00 + 90.00
Velcro wrist splint	One (1)	6.35
VACOped walker boot	One (1)	180.00

This online survey was distributed within the East Kent Hospitals Trust. Responses were included as valid entries if they fulfilled the inclusion criteria, which were as follows: response within the stipulated six-week time frame (October 14-November 30, 2022) and the participant working within a T&O department. To have control for the quantitative analysis, the actual costs of the items were sourced from the online East Kent Hospital Procurement Department [8]. Data analysis focused on comparisons between the actual and estimated price (average, median, and interquartile range (IQR)). An answer was deemed ‘correct’ if it was within 20% of the actual price. This was mimicked from previous studies to give a standardised results section [2].

## Results

**Figure 1:**
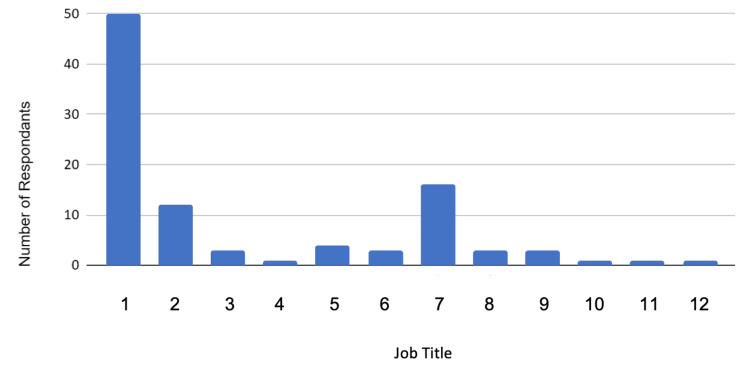
Distribution of survey respondents categorised by job title Job Titles: (1) Consultants, (2) Nurses, (3) Registrars, (4) Specialty Grade Doctor, (5) Associate Specialist, (6) Advanced Practice Physiotherapist, (7) Practitioners, (8) Coordinators, (9) Theatre Support Workers, (10) Day Surgery Manager, (11) Medical Secretary, (12) Theatre Audit Clerk

**Figure 2:**
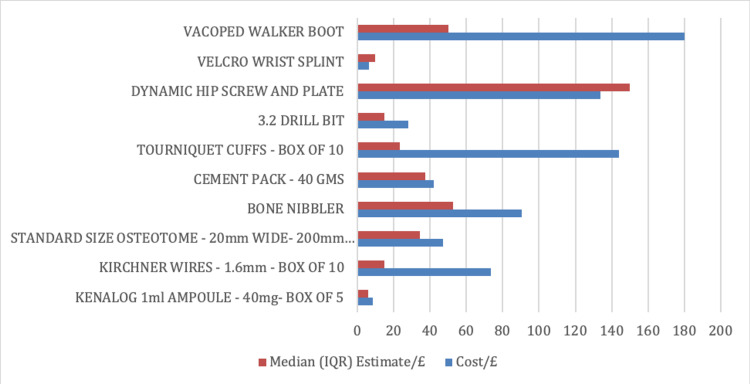
Accuracy of estimated median item costs compared to actual item costs

One hundred and five responses were recorded, with 98 being analysed after applying the inclusion/exclusion criteria. Demographic data are considered first. The distribution of job roles within the respondents is shown in Figure 1. Figure 2 gives a visual representation of the accuracy of estimated median costs compared to actual costs.

Table 2 shows job titles that were grouped together in Figure 1.

**Table 2:** Job titles that were grouped together

Distribution of Practitioners	Number of Respondents (% of total practitioners)
Theatre Practitioner	2 (12.5%)
Senior Orthopaedic Surgical Care Practitioner	1 (6.25%)
Orthopaedic Practitioner	2 (12.5%)
Scrub Practitioner	1 (6.25%)
Operating Department Practitioner	10 (62.5%)
Total Practitioners	16
Distribution of Coordinators	Number of Respondents (% of total coordinators)
Clinical Trauma Coordinator	1 (33.3%)
Orthopaedic Theatre Lead/Theatre Coordinator	1 (33.3%)
Theatre Emergency Coordinator	1 (33.3%)
Total Coordinators	3

The sex distribution of respondents was reported as 51 (52%) ‘Male’, 46 (47%) ‘Female’, and one (1%) ‘Preferring not to say’. Table 3 illustrates the following results. Approximately 7.1% of all item estimates were deemed ‘correct’. No correlation was seen between years of staff experience and the accuracy of estimates. Kenalog 1 mL ampoule (Kenalog, Bristol-Myers Squibb, NJ) had the highest accuracy of estimation across all responses with 13 (13%), respondents whilst both ‘kirschner wires’ and ‘3.2 drill bit’ had the lowest accuracy with only four (4% each) respondents correctly estimating the cost. The median estimated cost was closest to the actual cost for the ‘cement pack’ (median estimate/actual cost = 0.9). The median estimated cost was furthest from the actual cost for ‘tourniquet cuffs’ (median estimate/actual cost = 0.16). ‘Velcro wrist splint’ was the item that was the most overestimated (median estimate/actual cost = 1.57), with only two of the 10 items meaning overestimated (‘velcro wrist splint’ and ‘dynamic hip screw and plate’). The most underestimated item was ‘tourniquet cuffs’ (median estimate/actual cost = 0.16).

**Table 3:** Cost of each item, estimates, and percentage of correct estimates IQR: interquartile range

Item	Quantity	Cost/£	Median (IQR) Estimate/£	Median Estimate/Actual Cost	Percentage of Respondents Within 20%
Kenalog 1 mL ampoule	40 mg – box of 5	8.31	6.00 (12)	0.72	13
Kirschner wires - 1.6 mm	Box of 10	73.68	15.00 (42.25)	0.20	4
Standard size osteotome – 200 mm x 20 mm	One (1)	47.25	34.50 (62.00)	0.73	9
Bone nibbler	One (1)	90.56	52.50 (102.50)	0.58	6
Cement pack	40 grams	42.00	37.50 (72.50)	0.90	11
Tourniquet cuffs	Box of 10	144.18	23.50 (50.00)	0.16	5
3.2 mm drill bit	One (1)	28.00	15.00 (40.00)	0.54	4
Dynamic hip screw and plate	One (1)	44.00 + 90.00	150.00 (250.00)	1.12	7
Velcro wrist splint	One (1)	6.35	10.00 (20.00)	1.57	6
VACOped walker boot	One (1)	180.00	50.00 (75.00)	0.28	6

The question ‘Should the cost of an item influence its use?’ showed 60 (61.2%) participants answering with ‘Yes’ and 38 (38.8%) with ‘No’.

## Discussion

With the expenditure of the NHS overshadowing the growth of the country’s economy, the ever-surmounting financial pressure is significantly rising because of the cost-of-living crisis, following the pandemic. The NHS ‘Long Term Plan’ stipulates that the ‘key’ to ‘sustainable development and reducing the use of natural resources in line with government commitments’ lies in the ‘ reductions [of] single-use plastics, throughout the NHS supply chain’ [9]. With the ongoing drive to shift towards sustainable healthcare, the focus must be directed towards the utilisation of single-use disposable items. Hence, we conducted this survey to ascertain the cost-awareness of T&O staff, as this department is often under scrutiny for the overuse of consumables. Overall, this study shows a paucity of data pertaining to the cost-awareness of T&O specialists in the United Kingdom.

Only 7.1% of all estimates for all items were deemed ‘correct’, with the price of two out of 10 items in the questionnaire being overestimated. This indicates an underestimation of the cost of consumables and could be extrapolated to other equipment and services as well. If T&O staff believe that their equipment is cheaper than it is, then this suggests that there is an ability for intervention to have better outcomes both financially and economically. For example, if a department were to relate the cost or environmental impact to each misuse of a consumable, it may result in staff reconsidering their options when using consumables and consequently reduce overall waste and, therefore, money. This would be an example of a micro-allocation (varies from individual to individual). Considering the significance of macro-allocation (wider policies focussing on a wider scope), studies such as this one could highlight the disparities nationally between different specialities and create more specific plans for containing costs in relation to consumables [10].

One of the interventions employed currently in other countries in a bid to reduce wastage is price transparency [11]. Staff in these surgical specialities agreed that knowing the cost of items would cause them to reconsider their usage and, more importantly, wastage [12]. This is aligned with our study, where 60 (61.2%) participants replied with ‘Yes’ to the question ‘Should the cost of an item influence its use?’, suggesting that there is a need for this information to be distributed. However, certain methods of price transparency that may be fruitful in other countries may not work in the United Kingdom because of the public funding of the NHS. With the NHS treating all patients freely and equally at the point of care, this may have led staff to a feeling of non-responsibility pertaining to the cost of treatment, as it is not considered at the time of treatment and therefore not tangible. By reminding clinicians of their ethical duty not only through patient care but also through the effective use of taxpayer money and duty to the environment, it may be possible to decrease the impact of consumables.

To draw conclusions about the factor of experience in relation to cost awareness, the question ‘What is your job title?’ was asked and the time period the participants had been in the role; however, this did not account for previous experience before the current role respondents were in. This is a limitation of the study as the full extent of the subjects was not ascertained, and it is difficult to quantify as non-medical experience may also increase cost awareness. This is something that would be modified in future studies to allow full saturation of this factor in determining if experience had an effect on cost awareness.

The relevance of surveying all T&O staff must also be questioned. Whilst all medical professionals must have a basic duty towards reducing economic and environmental waste, doctors and nurses are often the ones who decide on which consumables to use. If health economics is taught throughout all healthcare curricula, but not applied in practice, it may lead to students adversely, giving health economics less importance because of being classed as ‘low yield’. This is a small tweak that can be made for future studies if it is deemed important to focus on a specific subgroup of staff.

The sample size was relatively small as it focused purely on the T&O department in one trust. This may not be a true representation of the national population we are trying to draw conclusions from and would need multiple trusts across the country to partake in this. It is difficult to link the success of education in varying curricula to job titles, as professionals come from a multitude of institutions, hospitals, and previous jobs.

## Conclusions

The awareness of the costing of equipment and price transparency to hospital staff in an attempt to combat the ever-rising financial strain have been key interventions highlighted by NHS England. Coupled with the global progression to sustainable healthcare (benefits patients and the environment, focussing on early intervention) being the golden standard, the usage and impact of disposable products must be scrutinised.

We hope this will provide a baseline for further studies in different geographical areas and initiate a push for better practice at national and trust levels.

## Appendices

**Table 4:** Questionnaire distributed to healthcare staff within the Trauma and Orthopaedic department

What is your age?
What best describes your gender?
What is your job title?
How long have you been working in this role?
Which NHS trust are you associated with?

One (1) 15 blade scalpel
One (1) 3-0 Vicryl (non-rapid)
One (1) 4-0 Prolene suture
One (1) 10 mL control handle syringe
One (1) package of two 4x4 sterile sponges
One (1) 1 L bag of intravenous normal saline
One (1) vial of 1.2g intravenous co-amoxiclav
One (1) box of fifty (50) surgical masks
One (1) plastic disposable skin stapler
One (1) disposable sterile surgical gown

Kenalog 1 mL ampoule
Kirschner wires, 1.6 mm
Standard size osteotome, 200 mm x 20 mm
Bone nibbler
Cement pack
Tourniquet cuffs
3.2 mm drill bit
Dynamic hip screw and plate
Velcro wrist splint
Vacoped walker boot
